# Dynamic Neural Network Modelling of Soil Moisture Content for Predictive Irrigation Scheduling

**DOI:** 10.3390/s18103408

**Published:** 2018-10-11

**Authors:** Olutobi Adeyemi, Ivan Grove, Sven Peets, Yuvraj Domun, Tomas Norton

**Affiliations:** 1 Engineering Department, Harper Adams University, Newport, Shropshire TF10 8NB, UK; oadeyemi@harper-adams.ac.uk (O.A.); igrove@harper-adams.ac.uk (I.G.); speets@harper-adams.ac.uk (S.P.); ydomun@harper-adams.ac.uk (Y.D.); 2 M3‐BIORES research group, Division of Animal and Human Health Engineering, Department of Biosystems, Katholieke Universiteit Leuven, Oude Markt 13, 3000 Leuven, Belgium

**Keywords:** irrigation scheduling, modeling, dynamic neural networks, soil moisture dynamics, sensors

## Abstract

Sustainable freshwater management is underpinned by technologies which improve the efficiency of agricultural irrigation systems. Irrigation scheduling has the potential to incorporate real-time feedback from soil moisture and climatic sensors. However, for robust closed-loop decision support, models of the soil moisture dynamics are essential in order to predict crop water needs while adapting to external perturbation and disturbances. This paper presents a Dynamic Neural Network approach for modelling of the temporal soil moisture fluxes. The models are trained to generate a one-day-ahead prediction of the volumetric soil moisture content based on past soil moisture, precipitation, and climatic measurements. Using field data from three sites, a $R^{2}$ value above 0.94 was obtained during model evaluation in all sites. The models were also able to generate robust soil moisture predictions for independent sites which were not used in training the models. The application of the Dynamic Neural Network models in a predictive irrigation scheduling system was demonstrated using AQUACROP simulations of the potato-growing season. The predictive irrigation scheduling system was evaluated against a rule-based system that applies irrigation based on predefined thresholds. Results indicate that the predictive system achieves a water saving ranging between 20 and 46% while realizing a yield and water use efficiency similar to that of the rule-based system.

## 1. Introduction

An increasing world population and climate change have placed a considerable amount of pressure on global freshwater supplies [1]. Irrigated agriculture is the world’s largest consumptive user of fresh water, accounting for over 70% of its global use [2]. It is therefore important to develop technologies which enable sustainable and efficient water use for irrigated agriculture while obtaining a healthy plant growth.

It is desirable to irrigate to meet specific plant water demands at the right time while avoiding over and under irrigation. This usually involves irrigation scheduling and control operations on an hourly or daily basis for a time period of usually less than a week [3]. Precision irrigation aims to accurately determine and quantify plant water needs. The irrigation amount and timing is based on measurements of soil, plant, and climatic variables from which the plant water need is inferred [4]. Precision irrigation has been shown to improve water use efficiency, reduce energy consumption, and enhance crop productivity by leveraging advances in sensor, control, and modelling technologies [5,6,7,8]. Such advances include the development of energy efficient and fault-tolerant wireless sensor networks [9,10], intelligent proximal sensing for the detection of plant water stress [11,12,13], and variable rate irrigation systems [6,14,15]. 

The temporal dynamics of field-scale soil moisture is perhaps the most leveraged tool for irrigation scheduling. This is because the soil moisture status is indicative of the water available for uptake by crops [16]. A number of irrigation scheduling methods estimate crop water needs using soil moisture and climatic data, and rules created by expert agronomists. Most of the commercial automated irrigation systems are programmed to irrigate at specific time intervals and apply a fixed irrigation volume. A number of these systems are also programmed to irrigate after a predefined soil moisture threshold is reached [17]. Due to their open-loop structure, these methods may not guarantee optimum irrigation scheduling decisions resulting in suboptimal plant health and efficiency in water use [18]. These shortcomings can be alleviated with the use of feedback control where sensor feedback is employed in optimizing irrigation timing and volume [19]. Although these approaches improve irrigation scheduling decisions, they do not include a model for the process dynamics; as a result, the overall system may not be robust to external perturbations [20].

Model-based irrigation scheduling systems consist of a calibrated internal model which employs feedback from soil, plant, and climatic sensors in order to predict crop water needs [20]. McCarthy et al. [21] implemented a model-based control system for predicting the irrigation requirements of cotton with an objective of maximizing crop yield. Their system relied on a complex crop model which requires detailed information on various climatic, soil and crop parameters. Park et al. [20] developed a model predictive control system for center pivot irrigation which used measured soil and climatic data to calibrate a complex soil-water model. The use of mechanistic models in these systems has many practical limitations because they are data demanding and require time-consuming calibrations during model development.

In recent years, many studies have investigated the applicability of data-driven machine learning models to irrigation decision support. Navarro-Hellín et al. [22] presented a regression model applied in predicting the weekly irrigation needs of a plantation using climatic and soil data as inputs. Giusti and Marsili-Libelli [23] applied a fuzzy decision system in predicting the volumetric soil moisture content based on local weather data. King and Shellie [24] used neural network modelling to estimate the lower threshold ($T_{nws}$) needed to calculate the crop water stress index for wine grapes. Delgoda et al. [25] applied a system identification model in predicting the soil moisture deficit using climatic and soil moisture data as model inputs. These statistical methods explore the spatial and temporal patterns hidden in historical data in order to map input data to an output space. They do not rely on a physical model of the system as they are data-driven (i.e., they learn from data). In many cases, these machine learning methods have been shown to achieve a good prediction performance [26]. They also have fewer data requirements when compared to mechanistic models [24,27,28].

For real-time irrigation scheduling, a model which is able to predict the soil moisture dynamics is desirable [18]. In order to achieve this with traditional machine learning and system identification methods, an extensive physical knowledge of vadose zone hydrology and boundary layer meteorology is required to derive robust input features from soil and climatic data. This is because of the complex nonlinear relationship between the climatic parameters, soil hydraulic properties, and the soil moisture dynamics [29]. Lozoya et al. [30], Delgoa et al. [25], Giusti and Marsili-Libelli [23], and Saleem et al. [31] presented system identification models for the prediction of soil moisture dynamics, which are parameterized based on the soil water balance method. This involved assumptions relating to the absence of surface run-off and deep percolation. The estimated evapotranspiration was also used as an input to the models. In practice, the true crop evapotranspiration may be significantly different from the estimated values. Furthermore, these models are only applicable to the site for which they are developed limiting their use for a different environment. This is because models developed using traditional machine learning and system identification approaches are mostly only applicable to the environment for which they were developed [22]. 

Machine learning approaches such as support vector machines (SVMs) and adaptive neuro-fuzzy inference systems (ANFIS) are another group of models that have been applied for the prediction of soil moisture dynamics [26,32,33,34]. These approaches have good prediction capability and limited input requirements. Karandish and Šimůnek [26] compared various machine learning models including ANFIS and SVM for simulating the time series of soil moisture content using meteorological, precipitation, and crop coefficient data as model input. The authors reported that the models achieved a prediction performance comparable to that of a mechanistic physical process-based model: HYDRUS–2D. However, they noted that these machine learning models are not suitable for the entire range of soil moisture prediction i.e., water stress conditions. It is therefore evident that robust and scalable data-driven models need to be developed for irrigation scheduling applications. 

Neural network (NN) methods have a strong learning ability and are able to represent the nonlinear relationship between the inputs and outputs of a system [35]. Some specific applications of neural networks to irrigation and water resource management include the prediction of soil moisture to aid irrigation scheduling [35,36], crop yield prediction [37,38], prediction of irrigation water demand [39,40], rainfall-runoff modelling [41,42] and groundwater modelling [43,44].

An NN method is applied in this study for predicting the soil moisture dynamics because of their ability to produce robust functions approximating complex processes [45]. However, traditional feedforward neural networks (FFNNs) have limited ability to model dynamic data because they are unable to preserve previous information, resulting in suboptimal predictions when they are applied in modelling highly causal systems [46]. The learning capability of FFNNs can be improved through additional pre-processing of dynamic data and combining the FFNN with other methods including genetic algorithms [47] and fuzzy logic [36]. For example, Pulido-Calvo and Gutiérrez-Estrada [40] applied a hybrid FFNN model to generate a one-day-ahead forecast of daily irrigation water demand. The forecast produced by the FFNN was corrected via a fuzzy logic approach whose parameters were adjusted using genetic algorithms. While this sort of hybrid modelling approach can strengthen the ability of an FFNN to learn dynamic data, the long-term generalization ability of such models is limited due to the ad hoc nature of fuzzy logic rules. Furthermore, the methods that employ additional pre-processing of dynamic data are time-consuming because of the extensive time and frequency domain computations they rely on. The data pre-processing steps also rely on subjective user intervention, which limits the scalability of the models to new environments.

This present study focuses on a dynamic modelling task, for which the Recurrent Neural Network (RNN) presents a suitable solution. An RNN has internal self-looped cells, allowing it to preserve information from previous time steps [48]. The Long Short-Term Memory Network (LSTM), a class of RNNs was selected for this study because of its successful application in the control of nonlinear dynamic systems [49,50]. The LSTM requires minimal input data pre-processing and is able to preserve useful information across multiple time steps [51]. They have been shown to achieve robust performance in modelling sequential data in fields such as natural language processing [52], time series classification of chaotic systems [53], and speech recognition [54]. Zhang et al. [55] demonstrated a hydrological application of LSTM models for the prediction of water table depth. Time series data on water diversion, evaporation, precipitation and temperature were applied as inputs to the model. The authors reported $R^{2}$ scores ranging between 0.789 and 0.952 for the LSTM models, largely outperforming FFNN models which achieved a maximum $R^{2}$ score of 0.495. The robust water table depth prediction achieved by the LSTM models highlights their ability to preserve and learn previous information from long-term time series data typical of hydrological application. This ability is particularly desirable in soil moisture based irrigation scheduling where the present soil moisture content is dependent on past soil moisture, precipitation, and climatic data.

The objective of this study was to develop LSTM models for the prediction of volumetric soil moisture content for three sites with different soil characteristics. Performance of the LSTM models was evaluated by comparing the LSTM predicted soil moisture content with measured soil moisture content and estimated soil moisture content using traditional feedforward neural networks (FFNNs). The applicability of the LSTM models for prediction in sites not used in model training was also investigated. Finally, the application of the LSTM models in predictive irrigation scheduling was demonstrated using model-based simulations of the potato-growing season.

The rest of the paper is structured as follows. In Section 2, the theoretical background of neural networks is presented. In Section 3, the methodology employed for the study is presented. Section 4 shows the performance evaluation of the neural network models and the predictive irrigation scheduling system. In Section 5, conclusions and recommendations for future work are presented. 

## 2. Background

This section presents a theoretical background on artificial neural networks including the FFNN and the recurrent neural network.

### 2.1. Neural Network Preliminaries

The basic building block of neural networks is the neuron. It is a processing element that takes a number of inputs, applies a weight to them, sums them up, includes a bias term, and passes the result to an activation function, which then produces an output. This activation function implements a nonlinear transformation to the linearly combined input in order to produce a nonlinear output.

Through a combination of these neurons across the input space and connections of the neurons outputs to other neurons, a function can be learned which maps the nonlinear relationship between an input feature space and an output target. The input–output relation of the system can be described by Equation (1) [45]:

(1) $$Z_{j}^{i}\left(k\right)=f\left(\overset{N}{\underset{i=1}{\sum }}w_{ij}^{i}x_{i}^{i-1}\left(k\right)+\;\delta^{i}\right)$$

where f(:) is the nonlinear activation function, and $w_{ij}^{i}$ is the connection weight of the *j*-th neuron unit in the (*i* − 1)-th layer to those of the *i*-th layer. $x_{i}^{i-1}$ is the input from the (*i* − 1)-th layer and $\delta^{i}$ are the respective bias terms.

### 2.2. The Feedforward Neural Network

**Figure 1:**
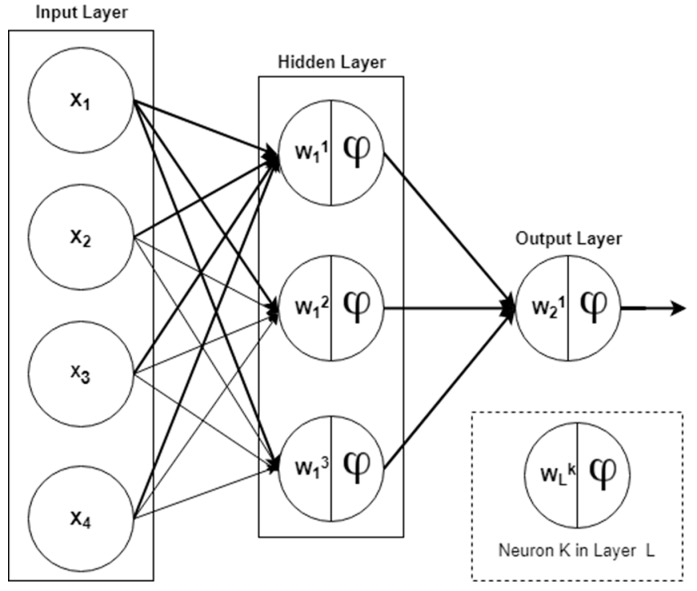
The feedforward neural network (FFNN).

The FFNN, also known as the multilayer perceptron (MLP) network, is built by ordering neurons in layers and letting each neuron in a layer take as input only the outputs of the units in the previous layer or external inputs. A network with N=1,2,3,…,n layers is called an n layer network. The FFNN is shown in Figure 1.

The second layer in Figure 1 is called the output layer as it produces the output of the network. The first layer is known as the hidden layer since it is located between the external inputs and the output layer. The mathematical formula expressing the FFNN is detailed in Equation (2) [45]

(2) $${\hat{y}}_{i}=g_{i}\left[x,\theta \right]=F_{i}\left[\overset{n_{h}}{\underset{j=1}{\sum }}\varphi_{i,j}f_{j}\left(\overset{n_{\varphi }}{\underset{l=1}{\sum }}w_{j,lxl}+w_{j,0}\right)+\varphi_{i,0}\right].$$

In Equation (2), θ is the parameter vector containing all the adjustable parameters of the network i.e., the weight and the biases $\left\{w_{j,l},\varphi_{i,j}\right\}$, and $f_{j}$ is the nonlinear activation function. The biases usually take a value of 1.

In order to determine the value of the weights, the network is trained with data containing examples of the inputs $x_{l}$ and outputs $y_{i}$ pairs, known as the training set. The weights are chosen to minimize a global loss function, which measures the cost of predicting $\hat{y}$ when the true output y is a function over the training set. For regression problems that encompass dynamic modelling tasks, the cost function to be minimized is the mean-squared error, which is computed as shown in Equation (3):

(3) $$l\left(\hat{y},y\right)=\;\overset{K}{\underset{k=1}{\sum }}E\left(k\right)=\frac{1}{2n}\overset{K}{\underset{k=1}{\sum }}\overset{n}{\underset{i=1}{\sum }}\|{\hat{y}}_{i}\left(k\right)-y_{i}{\left(k\right)\|}^{2}$$

where $l\left(\hat{y},y\right)$ is the loss function, and n is the number of training examples. The minimization of the loss function and update of weights is achieved using the backpropagation algorithm [56].

### 2.3. Long Short-Term Memory Network

The LSTM is a variant of the RNN, so it is expedient to introduce the RNN before describing the LSTM.

**Figure 2:**
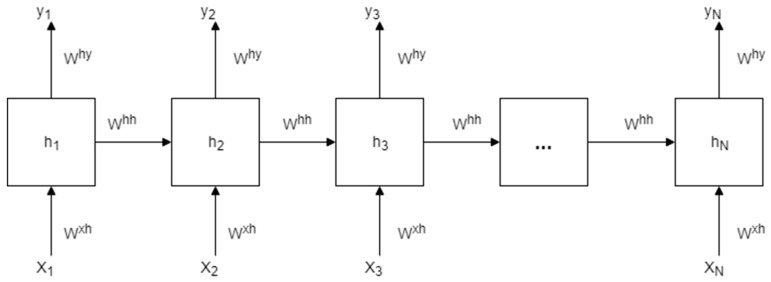
An unrolled recurrent neural network.

Recurrent neural networks are similar to FFNNs except that there is a self-feedback of neurons in the hidden layers as illustrated in Figure 2. This gives the network memory and it is able to learn from an entire sequence given portions of the overall sequence, i.e., it is a dynamic system.

The hidden nodes $h=\left(h_{1},\ldots \;\ldots ,\;h_{N}\right)$ and output nodes $y=\left(y_{1},\ldots \;\ldots ,\;y_{N}\right)$ are computed by looping through Equations (4) and (5) below [45]:

(4) $$h_{t}=\tan h\left(b_{h}+Wh_{t-1}+Ux_{t}\right)$$

(5) $$y_{t}=b_{o}+Vh_{t}$$

where $x_{t}$ is the input vector at time t, and $h_{t-1}$ is the hidden cell state at time t−1, $b_{o}$ and $b_{h}$ are the vectorised bias terms, and U,W,V are the weight matrices for input-to-hidden, hidden-to-hidden, and hidden-to-output connections, respectively.

The loss is calculated as the total loss for each time-step and the gradients are computed via back-propagation through time (BPTT) [57].

However, BPTT is not able to learn a pattern from long-term dependency because of the gradient vanishing problem [58]. The RNNs use their back-coupling connections to memorize short-term dependency in a sequence; as a result, the backpropagated error signals in time can become infinitely high or vanish [59]. Hochreiter and Schmidhuber [60] proposed the LTSM, which is able to solve the exploding or vanishing gradients problem by enforcing constant error flows through constant error carousels within special multiplicative units. These units regulate the error flow in the network by learning how to open or close specialized gates in the network. The constant error carousels (CECs) and the multiplicative and gate units form the memory block of the LSTM [55].

**Figure 3:**
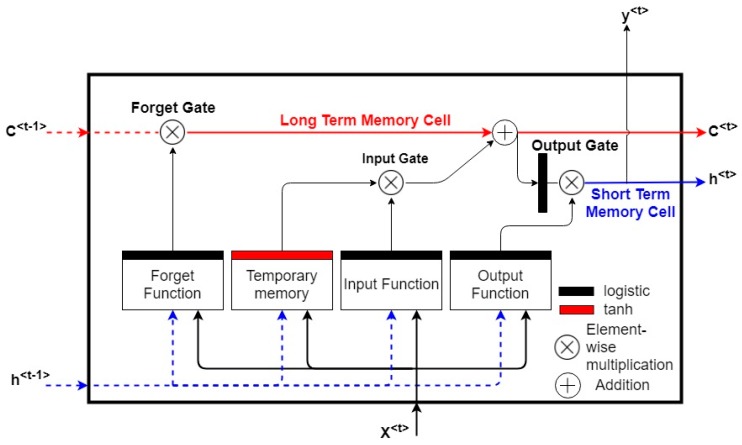
The long short-term memory (LSTM) network memory block.

The CEC loops through the network without an activation function, so the vanishing gradient problem does not occur when BPTT is applied to train an LSTM [45]. Therefore, LSTMs are able to approximate long-term information because the information can flow easily along the cells unchanged. The input, forget, and output gates of the memory block control the input into the CEC cell, the information retained in the cells, and the output from the cell into other blocks in the network. A schematic representation of the LSTM memory block along with its associated components is shown in Figure 3.

The LSTM computes the mapping from an input sequence x to the output by looping through Equations (6)–(11) with initial values $C_{o}=0$ and $h_{o}=0$ [45]:

(6) $$i_{t}=\sigma \left(w_{i}x_{t}+U_{i}h_{t-1}+b_{i}\right)$$

(7) $$f_{t}=\;\sigma \left(w_{f}x_{t}+U_{f}h_{t-1}+b_{f}\right)$$

(8) $$\sigma_{t}=\sigma \left(w_{o}x_{t}+U_{o}h_{t-1}+b_{o}\right)$$

(9) $${\tilde{C}}_{t}=\tan h\left(w_{c}x_{t}+U_{c}h_{t-1}+b_{c}\right)$$

(10) $$C_{t}=\;f_{t}⊗C_{t-1}+i_{t}⊗{\tilde{C}}_{t}$$

(11) $$h_{t}=o_{t}⊗\tanh\left(C_{t}\right)$$

where $w_{i},\;w_{f},\;w_{o}$ are the weight matrices from the input, forget, and output gates to the input, respectively, $U_{i},U_{f},\;U_{o}$ are the matrices of the weights from the input, forget, and output gates to the hidden layer, respectively, $b_{i},\;b_{f},\;b_{o}$ are the bias vectors associated with the input, forget, and output gates, σ is the nonlinear sigmoid activation function $\sigma \left(x\right)=\;\frac{1}{1+\;e^{-x}}$, and $i_{t},\;f_{t},\;o_{t},\;C_{t}$ are the input, forget, and output gate and the cell state vectors at time t, respectively. The element-wise vector multiplication is denoted with ⊗.

## 3. Methodology

The methodology employed for this study is presented in this section. This includes an overview of the data applied for developing the soil moisture prediction models, the structure of the neural network models, and the structure of the predictive irrigation scheduling system.

### 3.1. Study Sites and Data Source

The data applied in developing the neural network (NN) models for soil moisture prediction were obtained from three study sites, which are part of the Cosmic-Ray Soil Moisture Observing System (COSMOS) monitoring project in the United Kingdom [61]. Briefly, the COSMOS project is a soil moisture and climate monitoring network operating in the UK, USA, Australia, and China. The project provides near real-time soil moisture and climatic data for use in a variety of applications including agriculture, water resources management, flood prediction, and land-surface modelling.

The data obtained for the three study sites included hourly measurements of windspeed, rainfall, air temperature, net radiation, relative humidity, and volumetric soil moisture content. Details of the three sites are summarized in Table 1.

The volumetric soil moisture content in all sites is measured using the cosmic-ray soil moisture sensor (Model CRS-1000/B, Hydroinnova LLC, Albuquerque, NW, USA) deployed using a site-specific calibration. The cosmic-ray soil moisture sensor consists of a non-invasive probe which measures the neutron emitted by cosmic rays within the air and soil. These neutrons are moderated by hydrogen atoms emitted from soil water into the atmosphere. The neutrons and hydrogen atoms combine instantaneously and its density is inversely correlated with soil moisture [62]. A calibration function defines the relationship between the neutron intensity and soil moisture. This calibration function is simple, monotonic, and invariant with soil texture and chemical composition [63]. The sensor has a horizontal measurement range of 200 m and an effective measurement depth of up to 60 m. The sensor is reported to have an accuracy of ±2% measured volumetric soil moisture content [64]. Full details on the operating principle of the sensor can be found in Shuttleworth et al. [61]. The meteorological variables (e.g., air temperature, relative humidity, net radiation, windspeed, and precipitation) in all sites are measured by a MetPak Pro Base automatic weather station (Gill Instruments, Hampshire, UK). 

The NN models trained on data from the sites listed in Table 1 were also applied in predicting the soil moisture content in two independent sites with soil characteristics similar to that of the sites for which the models were trained. This was done to evaluate the applicability of the models for prediction in new sites, which were not used in model training. A summary of the independent sites is presented in Table 2.

### 3.2. Data Cleaning and Pre-Processing

The hourly data was resampled to daily (24 h) intervals as this is a time period applicable for field scale irrigation scheduling [65]. The daily averages of the climatic variables were calculated during the resampling, while the daily precipitation was calculated as the sum of daily rainfall and irrigation depths. The volumetric soil moisture content was also resampled to its average daily value. The data cleaning steps included imputing of missing values and removal of outliers.

**Figure 4:**
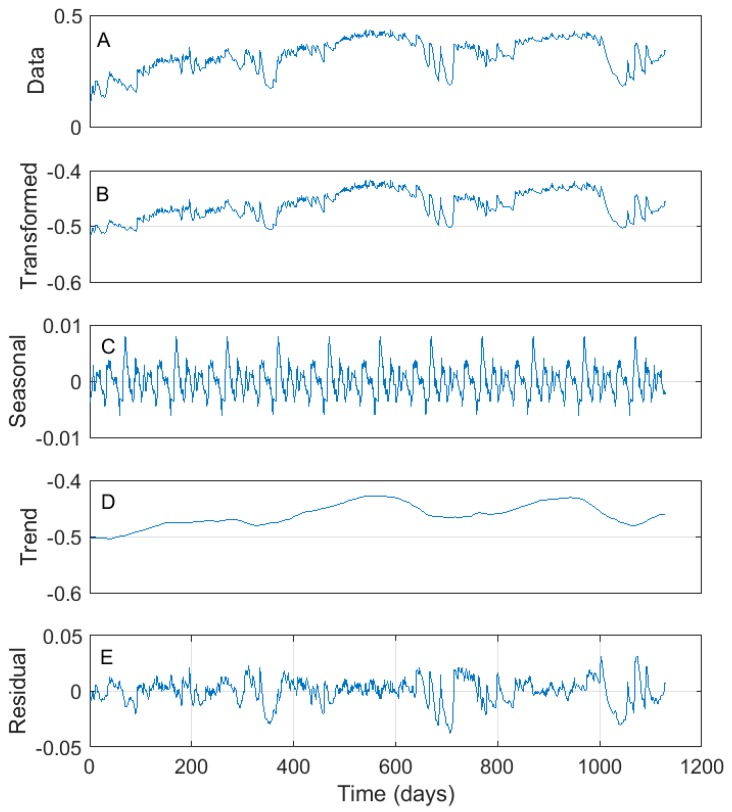
Soil moisture data transformation and decomposition prior to modelling. (**A**) Observed data. (**B**) Box–cox transformed data. (**C**) Seasonal component. (**D**) Trend component. (**E**) Residual component.

The pre-processing steps applied for the data modelled with the FFNN included a box–cox transform [66] of the soil moisture and air temperature data in order to stabilize their variance. The transformed data were thereafter deseasonalized using the seasonal and trend decomposition using loess (STL), as proposed by Cleveland et al. [67]. Several studies have shown that deseasonalizing dynamic data that exhibits seasonality prior to modelling is necessary in order to produce robust predictions with an FFNN model [68,69]. The STL technique decomposes the soil moisture and air temperature data into their trend, seasonal, and residual components. Thereafter, the sums of the trend and level were passed to the next step of the data pre-processing. An example of the transformed and decomposed soil moisture data is shown in Figure 4. In the next data pre-processing step, the climatic, precipitation, and soil moisture data were standardized by computing the z-score of their data points. In the post-processing stage, the soil moisture predictions were back-transformed to their actual scale through an inverse z-score transformation, the addition of the seasonal component, and an inverse box–cox transformation.

For the LSTM, the only data pre-processing step applied was a standardization of the climatic, precipitation, and soil moisture data. This was accomplished by computing the z-score of their data points. In the post-processing stage, the soil moisture predictions were back-transformed to their actual scale through an inverse z-score transformation.

For the model training sites, the dataset was divided into a 70:30 ratio for the purpose of model training and evaluation. The division was done such that the temporal nature of the data was accounted for; i.e., the evaluation dataset was posterior to the training dataset.

Data spanning 2016–2017 for the independent sites (Table 2) was applied in evaluating the prediction performance of the trained NN models on those sites.

### 3.3. The Proposed Model Framework

For predictive irrigation scheduling, a one-day-ahead prediction of the soil moisture content is required. The soil moisture content at time t+1 is a nonlinear function of past and present climatic, and precipitation inputs. It is also influenced by the past and present soil moisture content values. This is a Multiple Input and Single Output (MISO) system. The FFNN and LSTM networks are encoded in various suitable architectures appropriate for the learning task. The neural networks were developed using the Keras Deep Learning library on the Python programming platform [70].

#### 3.3.1. The Feedforward Neural Network Structure

The FFNN is straightforward to employ for discrete time modelling of dynamic systems for which there is a nonlinear relationship between the system’s inputs and output. The soil moisture dynamics can be modelled as a Nonlinear Autoregressive with Exogenous Input System (NARX) as shown in Equation (12):

(12) y(t+1)=S[y(t),…,y(t−j),u(t),…,u(t−n),p(t),…,p(t−m)]

where y(t+1) is the one-day-ahead prediction of the volumetric soil moisture content, y(t=0…j) are the present and past soil moisture content at day t=0…j, u(t=0…n) are the climatic inputs at day t=0…n, p(t=0…m) are the precipitation inputs at day t=0…m, and S is a nonlinear function, which is approximated using the FFNN.

The time lags m, n, and j  are determined through experimentation. The number of hidden layers in the network and the number of neurons in each hidden layer are also determined through experimentation. The soil moisture prediction is framed as a regression problem; as such, an appropriate activation function is required for the hidden layers of the FFNN. For regression problems, the most robust nonlinear activation function is the point-wise rectified linear units (RELU),max(0,x), where x is the input into the neuron. The RELU activation function is reported to provide easier optimization, faster convergence, and better generalization with the added bonus of being computationally efficient [71].

During the study, the RELU nonlinearity was applied in the hidden layers, while the network loss was minimized using the adaptive moment estimation (ADAM) optimization algorithm, which is reported to improve network convergence [72].

#### 3.3.2. The Long Short-Term Memory Network Structure

For modelling dynamic systems, the LSTM introduces a nonlinearity from the input to system states followed by a dynamic linearity from the states to the output. This can be represented in the state space form as shown in Equations (13a) and (13b):

(13a) x(t+1)=NNi[x(t)…x(t−k),u(t)…u(t−n),y(t)…y(t−j),p(t)…p(t−m);V]

(13b) y(t+1)=NNo[x(t+1);W]

where x(t+1) is the future state of the network at day t+1, x(t=0…k) are the present and past network states at day t=0…k, y(t=0…j) are the present and past soil moisture content at day t=0…j, u(t=0…n) are the climatic inputs at day t=0…n, p(t=0…m) are the precipitation inputs at day t=0…m, and y(t+1) is the one-day-ahead prediction of the volumetric soil moisture content. V is the parameter set of the network that corresponds to the states, and W is the parameter set of the network that corresponds to the output.

The time lags m, n, and j are determined through experimentation while the time delay k for the states is learned implicitly by the network during training. The network is designed as an LSTM nonlinear element (NNi) followed by a linear output layer (NNo). The number of LSTM layers and the number of memory blocks in each layer are also determined through experimentation. During the study, the network loss was minimized using the ADAM optimization algorithm.

### 3.4. Irrigation Scheduling

A predictive irrigation scheduling system is enabled by a model that uses feedback from soil and climatic sensors to predict the crop water demand [20]. A trained neural network model is able to generate soil moisture predictions and presents an opportunity for implementing predictive irrigation scheduling.

In order to demonstrate the applicability of a trained LSTM for predictive irrigation scheduling, the AQUACROP model developed by the Food and Agricultural Organization was used in simulating soil–plant–atmosphere interactions for the potato crop [73,74,75]. The AQUACROP model has been widely validated and is able to simulate soil moisture dynamics and crop response to water deficits across various soil types as a function of climatic inputs and water availability [76,77,78,79].

Climatic and rainfall data for the model training sites were used as inputs into the AQUACROP model. The LSTM models trained for each site was applied in generating a one-day-ahead prediction of soil moisture content using the climatic data and AQUACROP simulated soil moisture as inputs. Thereafter, the prediction was used to determine the irrigation depth and timing during the AQUACROP simulations. This formed the basis of the predictive irrigation system described in Section 3.4.1. The AQUACROP soil file was modified to represent the soil types and characteristics for the model training sites as summarized in Table 3. The crop characteristics of the default Lima potato file was used during the simulations.

The predictive irrigation system was compared to a rule-based irrigation scheduling system set up on AQUACROP. The rule-based system was programmed to apply irrigation based on specified soil moisture thresholds and applied water depths to refill the soil moisture content to field capacity. It was set up as an open-loop system, which does not consider soil moisture feedback after irrigation events. It should be noted that only data from the evaluation dataset set of the model training sites was applied in the simulations.

#### 3.4.1. Predictive Irrigation Scheduling System

The goal of irrigation scheduling is to maintain the soil moisture content between an upper and lower bound. The upper bound is usually defined as the field capacity while the lower bound is a point above the permanent wilting point expressed a function of the management allowable depletion (*MAD*).

In irrigation, it is common practice to express the amount of water retained in the plant root zone ($W_{r}$) as an equivalent depth of soil water (mm of water). This is expressed as shown in Equation (14):

(14) $$W_{r}=1000\theta Z_{r}$$

where θ is the volumetric soil moisture content, and $Z_{r}$ is the thickness of the root zone in meters.

The water deficit at time t ($DP_{t}$) is expressed as shown in Equation (15):

(15) $$DP_{t}=W_{r,FC}-W_{r,t}$$

where $W_{r,FC}$ is the water depth at field capacity, and $W_{r,t}$ is the water depth at time t. It is evident from Equation (15) that the water deficit at the upper bound ($DP_{U}$) will be zero, i.e., ($W_{r,FC}-W_{r,FC}$). The deficit at the lower bound ($DP_{L}$) is determined from a knowledge of the soils available water and the crops *MAD*. This is expressed as shown in Equations (16a) and (16b)

(16a) $$DP_{L}=W_{r,FC}-W_{r,LB}$$

with

(16b) $$W_{r,LB}=W_{r,FC}-MAD\left(W_{r,FC}-W_{r,PWP}\right)$$

where $W_{r,LB}$ is the water depth at the lower bound, and $W_{r,PWP}$ is the water depth at permanent wilting point. $DP_{L}$ will vary over the growth season as a result of root growth.

**Figure 5:**
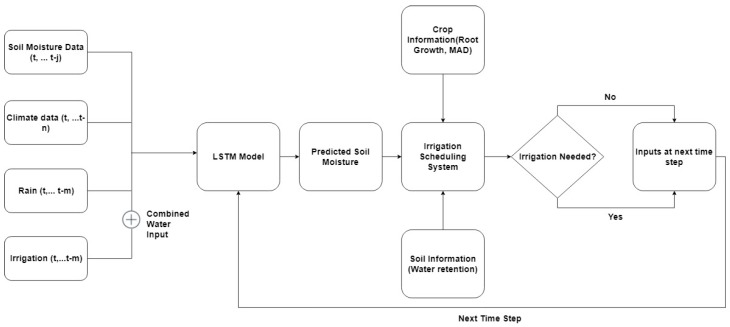
Block diagram of the predictive irrigation scheduling system. t is the time in days, m, n, and j are past time steps.

If a prediction of the soil volumetric soil moisture content at time t+1 is available from the LSTM model, the deficit at time t+1 ($DP_{t+1}$) can be easily calculated. The irrigation amount is computed as the water application depth that will replenish the water deficit to the upper bound i.e., Irrigation = ($DP_{t+1}$). For close-loop irrigation scheduling, the irrigation threshold is set at a safe point below $DP_{L}$. The advantage of this simplified irrigation scheduling system is the inclusion of a time variable lower bound. Delgoda et al. [65] noted that this is difficult to achieve with the optimization schemes applied in model predictive control systems. A block diagram of the proposed irrigation scheduling system is presented in Figure 5. Figure 5 shows that soil moisture, precipitation, irrigation, and climatic data are applied as inputs into a trained LSTM model in order to generate a prediction of the soil moisture content. The predicted soil moisture content is then used in conjunction with information on crop water requirement and soil water retention to determine the irrigation timing and amount.

During the simulations, for both the predictive and rule-based irrigation scheduling system, a *MAD* of 30% was assumed for the potato crop and the lower bound was dynamically adjusted as a function of rooting depth growth during the simulated growing season.

### 3.5. Model Evaluation Criteria

To assess the performance of the trained models for the prediction of the soil moisture content during the model evaluation, several measures of accuracy were applied. The model’s accuracy between the observed and predicted soil moisture content was evaluated using the coefficient of determination ($R^{2})$, root mean squared error (RMSE), and the mean absolute error (MAE).

The $R^{2}$ describes the proportion of the total variance in the observed data that is explained by the model and ranges between [−∞,1]. A $R^{2}$ close to 1 indicates that the model explains well the variance of observations. It is expressed as $R^{2}$ In Equation (17):

(17) $$R^{2}=\frac{\sum_{i=1}^{N}{\left(y_{i}-\bar{y}\right)}^{2}-\sum_{i=1}^{N}{\left(y_{i}-{\hat{y}}_{i}\right)}^{2}}{\sum_{i=1}^{N}{\left(y_{i}-\bar{y}\right)}^{2}}$$

where $y_{i}$ is the measured value at time i, $\bar{y}$ is the mean of $y_{i}$ (i=1…N), and ${\hat{y}}_{i}$ is the predicted value at time i.

The RMSE and MAE quantify the prediction errors in the same units of the variables. However, the RMSE strongly penalizes large outliers; as such, it is preferable to compliment it with the MAE [80]. RMSE and MAE values close to zero indicate good model predictions. The *MAE* and *RMSE* are defined as

(18) $$MAE=\frac{1}{n}\overset{n}{\underset{i=1}{\sum }}\left|y_{i}-{\hat{y}}_{i}\right|$$

(19) $$RMSE={\left[\frac{\sum_{i=1}^{n}{\left(y_{i}-{\hat{y}}_{i}\right)}^{2}}{n}\right]}^{0.5}.$$

In Equations (18) and (19), $y_{i}$ and ${\hat{y}}_{i}$ are observed and predicted value at time i (i=1,2,…,n), respectively.

## 4. Results and Discussion

The structure of the neural network models, their predictive performance, and the performance of the predictive irrigation scheduling system are presented and evaluated in this section.

### 4.1. Model Structure

The model structure and hyper-parameters of the neural network (NN) models were determined through a five-fold cross-validation on the training dataset. The model structures which achieved the best performance for the one-day-ahead prediction of the soil moisture content across the different sites are summarized in Table 4.

Table 4 shows that a first-order model taking the precipitation, climatic variables, and soil moisture content at the present day as inputs is able to predict the soil moisture content of the next day for the sandy loam (Baluderry) and loam (Stoughton) sites. For the heavier textured clay site (Waddeston), the soil moisture content at the next day is dependent on the precipitation and soil moisture during the present and previous day. This can be explained by the low infiltration capacity of heavier textured soils. The precipitation input on any day may take a time period greater than a day to completely infiltrate the soil column.

It was also found that a single layer of neurons and memory blocks in both the FFNN and the LSTM is able to satisfactorily model the soil moisture dynamics across all the sites. Additional layers could not further improve the learning capabilities of both networks. As an example, the performance of NN models with the same model structure with those listed in Table 4 but with two hidden layers are presented in Table 5. The two-layer models achieve a lower prediction accuracy across all sites. Moreover, as part of the model training experiments, the best cross-validation performance achieved by an FFNN, which included only a z-score transformation of the modelled data was an $R^{2}$ value of 0.68.

### 4.2. Soil Moisture Content Prediction

The prediction capability of a model is exemplified by its performance on data not seen by the model during training. As such, the prediction capability of the models was tested on the evaluation dataset set aside for each of the model training sites.

The prediction performance of a non-machine learning baseline which predicts the soil moisture content at a particular day as the average soil moisture content of the three previous days is presented in Table 6. This is presented along with the prediction performance of the trained NN models. A model will only be accepted as skillful if its performance surpasses that of the non-machine learning baseline. This is considered a good practice for approaching predictive modelling tasks [81].

Table 6 shows that both the FFNN and LSTM outperform the non-machine learning (naïve) baseline across all the sites. Therefore, the NN models can be accepted as being skillful. The FFNN and LSTM models are also shown to achieve a comparable prediction performance across all sites. However, it is interesting that the LSTM achieves a comparable performance to the FFNN without extensive pre-processing of input data. This highlights the ability of the LSTM to sufficiently learn the underlying function approximating dynamic data [51]. This ability is particularly desirable because the data pre-processing pipeline applied for the FFNN required subjective human intervention, which may not lead to an improvement in model performance for more complex dynamic systems.

**Figure 6:**
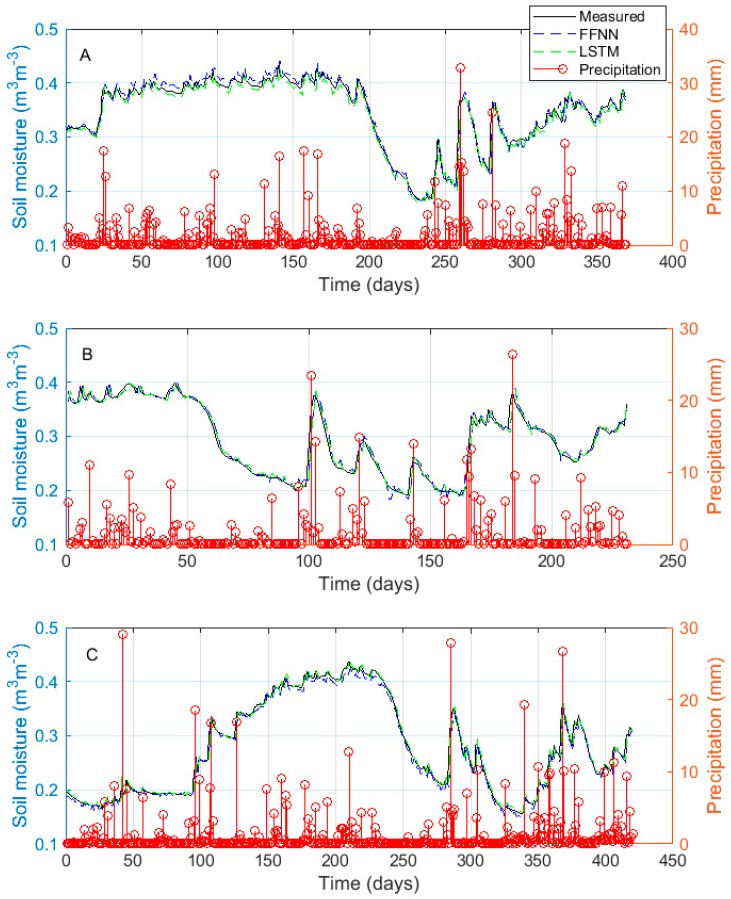
Measured soil moisture content and soil moisture content predicted by the FFNN and the LSTM using the evaluation dataset for the three training sites, (**A**) Baluderry, (**B**) Stoughton, and (**C**) Waddeston.

The soil moisture predicted by the FFNN and LSTM models along with the observed soil moisture content for the evaluation dataset is presented in Figure 6. Figure 6 shows that the LSTM models are able to accurately model the soil moisture dynamics while capturing its dominant modes. The LSTM models are also able to respond to perturbation from the precipitation input shown in the stem plots. Again, it is clear that the LSTM model is able to achieve a performance comparable to that of the FFNN with minimal input data pre-processing.

There have been previous attempts in literature to model the soil moisture dynamics and predict the soil moisture content in order to aid irrigation scheduling. Delgoda et al. [25] presented a linear dynamic model with assumptions made on the absence of saturation flows. This lead to a degradation in the modelling results. The saturation flows are a nonlinear function of the soils hydraulic properties [29]. The LSTM models presented in this study are able to implicitly learn such nonlinear relations during training. This is done during the adjustment of the network weights in order to define a function relating the climatic and precipitation inputs to the soil moisture content. Since soil moisture depends on the balance between water input and output, saturation flows have been incorporated in the LSTM model. Lozoya et al. [30] highlighted the need to parametrize several linear dynamics models for the prediction of soil moisture content for any particular site. This was attributed to the differing dynamics at saturation, the available water content, and that the permanent wilting point was not reached. The LSTM models are able to model these nonlinearities for the entire range of a site’s soil moisture content. The use of a single model for the entire range of operation of a process is usually favored for decision support purposes because of the need to ensure simple debugging and test procedures [82]. This may become complex when several models are used as part of a decision support system. This gives further evidence in favor of the application of the LSTM models for the purpose of soil moisture prediction and irrigation scheduling.

### 4.3. Prediction Performance in the Independent Sites

For the purpose of irrigation scheduling, it may be necessary to predict the soil moisture content for a new site for which historical data required to train an NN model is not available. The predictions will be generated using the climatic and soil variables of the new site as input into a model trained exclusively with data from another site. As such, the ability of the LSTM models to generate soil moisture predictions for independent sites using models from the training sites was evaluated. The prediction performance of the trained LSTM and FFNN models for these independent sites is presented in Table 7.

Table 7 shows that the LTSM models generate accurate predictions for the independent sites, and these predictions outperform those generated by the FFNN in terms of $R^{2}$ scores. This is because of the dynamic nature of the LSTM, which enables it to generate predictions as a function of model inputs and state maintained for a learned past time period. Table 7 also shows that the FFNN is unable to achieve a good prediction performance when the model trained in Baluderry was applied for prediction in Independent Site 1. This may be because the data pre-processing steps applied on the training data were not applicable to the data of Independent Site 1. This further highlights the robustness of the LSTM model, which is able to sufficiently learn the underlying function approximating the dynamic data. The data in Table 7 demonstrates the excellent approximation ability of the LSTM, which makes them useful for generating prediction for processes with an underlying dynamics similar to the process they were trained on. This approximation ability of the LSTM has been widely exploited in the field of time series forecasting where a single LSTM model is trained to predict data points for a time series belonging a common cluster [83].

The applicability of data-driven models trained for a particular site for prediction in a different site will further enhance the precision water management of various crops. Navarro-Hellín et al. [22] showed that models that are able to generalize to new sites not included in the model development are difficult to realize using traditional machine learning methods. The excellent generalization ability of the LSTM presents an opportunity for the development of multi-site soil moisture prediction models as demonstrated by the robust performance of the LSTM models presented in this study when tested on the independent sites.

### 4.4. Application in Predictive Irrigation Scheduling

**Figure 7:**
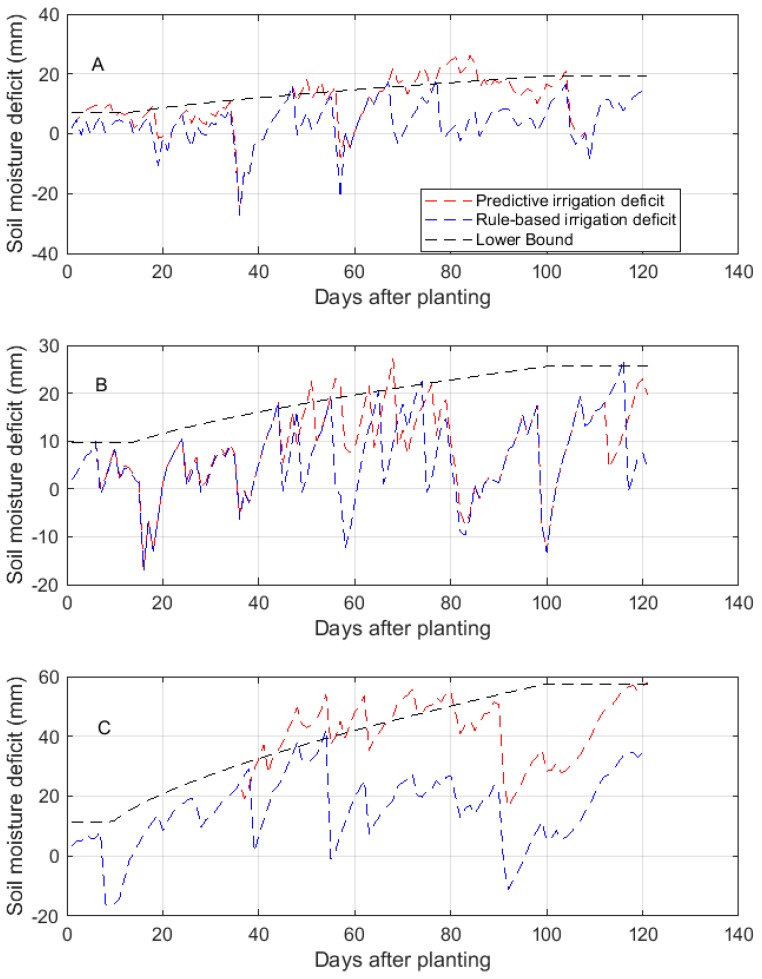
The predictive and rule-based irrigation scheduling systems for AQUACROP simulations of the potato-growing season on the three model training sites. (**A**) Baluderry, (**B**) Stoughton, and (**C**) Waddeston.

In this study, the purpose of modelling the soil moisture dynamics is to generate predictions of the volumetric soil moisture content which is required for predictive irrigation scheduling. As such, the LSTM model developed for each of the training sites was applied as part of a predictive irrigation scheduling system, which was evaluated alongside a rule-based system using AQUACROP simulations of the potato-growing season. The resulting soil moisture deficit for the predictive and rule-based systems, and the lower bound deficit during simulations for the three training sites are shown in Figure 7.

It should be noted that the negative deficit values in Figure 7 indicate soil moisture values above the field capacity, hence overwatering. Figure 7 shows that, in the heavy textured clay site (Waddeston), the predictive system violates the lower bound threshold during the mid-growing season. This occurred because the LSTM model applied in predicting the soil moisture content was not trained specifically for the potato crop. The high mid-season water demand of potato altered the dynamics learned by the model. Nevertheless, it can be seen that the deficits are close to the lower bound threshold and they are later minimized. It can also be seen that, across all sites, the rule-based system tends to over-irrigate, as indicated by the negative deficit values. Overall, the predictive system is able to maintain the soil moisture deficits within the allowable range and is able to account for the change in crop water requirements over the growing season.

The total water applied during the growing season along with the simulated crop yield and water use efficiency (WUE) is summarized in Table 8.

Table 8 shows that the predictive system consistently applied lower irrigation depths when compared with the rule-based system. The predictive system achieved a water saving of 46% in Baluderry, 20% in Stoughton, and 31% in Waddeston. The predictive system also achieved a yield and water use efficiency (WUE) similar to that of the rule-based system. These results confirm that the predictive system is suitable for irrigation scheduling and is able to improve water conservation.

## 5. Conclusions

The precise water management of crops will immensely benefit from automated decision support systems that integrate climatic and soil moisture measurements with a robust data-driven model of the soil moisture dynamics. This technology development will facilitate the prediction of crop water needs and an improvement in water conservation.

This paper has presented a dynamic neural network approach for modelling the time series of soil moisture content. The performance of the LSTM for the prediction of soil moisture content was evaluated for three sites with different soil characteristics. Using an independent evaluation dataset, the LSTM models developed for the sites achieved accuracies ($R^{2}>0.94$) for a one-day-ahead prediction. The LSTM models also generated accurate soil moisture predictions for independent sites not used in training the models.

The use of the LSTM models in predictive irrigation scheduling was also demonstrated using AQUACROP simulations of the potato-growing season. The performance of the proposed predictive irrigation scheduling system was evaluated by comparing its irrigation policies to those of a rule-based system. The predictive system was able to maintain the soil moisture deficit within allowable limits for the most part of the simulated growing season while minimizing over-irrigation. Furthermore, the predictive system was able to achieve a yield and WUE similar to that achieved by the rule-based system using lower irrigation application depths.

For future research, the predictive system should be extended to include rainfall forecasts. This will ensure that irrigation is optimized to further increase water savings through the maximum utilization of forecasted rainfall depths. The development of crop-specific LSTM models trained on a rich dataset obtained from sites with similar soil types will enhance the adoption of data-driven soil moisture models for use in irrigation scheduling applications.

## Figures and Tables

**Table 1:** Details of the sites used for model training.

Site Name	Soil Type	Land Cover	Date Range
Baluderry	Sandy loam	Farmland	May 2014–September 2017
Stoughton	Loam	Arable	August 2015–September 2017
Waddeston	Clay	Grassland	December 2013–September 2017

**Table 2:** The independent sites corresponding to each model training site.

Training Site		Independent Site 1			Independent Site 2	
	Name	Land Cover	Soil Type	Name	Land Cover	Soil Type
Baluderry	Bunny Park	Arable	Sandy loam	Bickley Hall	Grassland	Sandy loam
Stoughton	Morley	Arable	Loam	Cockle Park	Grassland	Loam
Waddeston	Hollin Hill	Grassland	Clay	Chimney Meadows	Grassland	Clay

**Table 3:** Soil characteristics of the model development sites applied in the AQUACROP simulation.

Site	$\mathrm{Field}\;\mathrm{Capacity}\;(m^{3}m^{-3})$	$\mathrm{Permanent}\;\mathrm{Wilting}\;\mathrm{Point}\;(m^{3}m^{-3})$	Profile
Baluderry	0.22	0.10	Sandy loam
Stoughton	0.31	0.15	Deep uniform loam
Waddeston	0.33	0.138	Clay

**Table 4:** The identified model structure with the best one-day-ahead prediction performance across the training sites.

Site	Model	FFNN					LSTM					
	N	M	J	Neurons	Layers	$R^{2}$	N	M	J	Blocks	Layers	$R^{2}$
Baluderry	1	1	1	40	1	0.95	1	1	1	20	1	0.95
Stoughton	1	1	1	20	1	0.97	1	1	1	20	1	0.97
Waddeston	1	2	2	20	1	0.99	1	2	2	40	1	0.99

N is the time lag associated with the climatic inputs, M is the time lag associated with the precipitation input, and J is the time lag associated with the past soil moisture content input.

**Table 5:** Training cross-validation performance of two-layer neural network models.

Site	FFNN	LSTM
	$R^{2}$	$R^{2}$
Baluderry	0.93	0.91
Stoughton	0.92	0.95
Waddeston	0.95	0.97

**Table 6:** Prediction performance of the non-machine learning (naïve) and neural network models when tested on the evaluation dataset for all the model training sites.

Site	Model	Naive			FFNN			LSTM	
	$R^{2}$	MAE $\left(m^{3}m^{-3}\right)$	RMSE $\left(m^{3}m^{-3}\right)$	$R^{2}$	MAE $\left(m^{3}m^{-3}\right)$	RMSE $\left(m^{3}m^{-3}\right)$	$R^{2}$	MAE $\left(m^{3}m^{-3}\right)$	RMSE $\left(m^{3}m^{-3}\right)$
Baluderry	0.89	0.02	0.03	0.94	0.01	0.01	0.95	0.01	0.01
Stoughton	0.88	0.02	0.03	0.97	0.01	0.01	0.97	0.01	0.01
Waddeston	0.92	0.01	0.02	0.99	0.01	0.01	0.99	0.01	0.01

**Table 7:** Prediction performance of the neural network models for the independent sites.

		Independent Site 1			Independent Site 2		
Models	Training Site	$R^{2}$	MAE $\left(m^{3}m^{-3}\right)$	RMSE $\left(m^{3}m^{-3}\right)$	$R^{2}$	MAE $\left(m^{3}m^{-3}\right)$	RMSE $\left(m^{3}m^{-3}\right)$
FFNN	Baluderry	0.74	0.04	0.07	0.93	0.01	0.01
	Stoughton	0.94	0.01	0.01	0.96	0.01	0.01
	Waddeston	0.95	0.01	0.01	0.94	0.01	0.01
LSTM	Baluderry	0.92	0.01	0.01	0.98	0.01	0.01
	Stoughton	0.96	0.01	0.01	0.98	0.01	0.01
	Waddeston	0.98	0.01	0.01	0.97	0.01	0.01

**Table 8:** Total irrigation application depth along with the simulated crop yield and water use efficiency for the potato growing season.

Site	Total Irrigation (mm)		Yield (ton/ha)		$\mathrm{WUE}\;(kgm^{-3})$	
	Predictive system	Rule-based system	Predictive system	Rule-based system	Predictive system	Rule-based system
Baluderry	69.50	129.80	12.64	12.64	4.08	3.93
Stoughton	141	177.20	12.64	12.64	3.68	3.68
Waddeston	55	79.90	12.64	12.64	3.82	3.85
